# mHealth resources to strengthen health programs

**DOI:** 10.9745/GHSP-D-14-00013

**Published:** 2014-02-11

**Authors:** Kelly L'Engle, Laura Raney, Margaret D'Adamo

**Affiliations:** a FHI 360, Durham, NC, USA; b FHI 360 for the Knowledge for Health Project, Baltimore, MD, USA, Now with Jhpiego, Baltimore, MD, USA; c U.S. Agency for International Development, Washington, DC, USA

## Abstract

A suite of resources provides implementation guidance for mHealth initiatives, particularly in less developed countries. The suite includes an eLearning course, online guide, evidence database, and a High-Impact Practices brief, along with the mHealth Working Group and website.

We note with interest that the article “mHealth innovations as health system strengthening tools: 12 common applications and a visual framework,” by Labrique et al.^1^ is the most viewed article in *Global Health: Science and Practice* to date. We believe this is indicative of the high level of interest in using mobile technology to strengthen health service delivery and to expand the reach of health promotion activities in countries around the world.

We would, therefore, like to introduce your readers to a suite of mHealth resources developed by the U.S. Agency for International Development (USAID) and its partner, the Knowledge for Health (K4Health) Project, with input from FHI 360 and the mHealth Working Group. The resources are designed to strengthen mHealth capacity of health program implementers and managers, particularly in less developed countries.

First, we have developed a new online eLearning course as part of USAID's Global Health eLearning Center (www.globalhealthlearning.org/course/mhealth-basics-introduction-mobile-technology-health). This self-directed course, **mHealth Basics: Introduction to Mobile Technology for Health**, explains the potential uses, benefits, and limitations of mHealth. It also highlights some existing applications, draws some preliminary conclusions from the evidence, and shares recommended best practices for mHealth program planning, design, monitoring and evaluation, scale up, and sustainability. It takes about 3 hours to complete the course.

**The mHealth Planning Guide: Key Considerations for Integrating Mobile Technology into Health Programs** is an online guide to help global health practitioners implement mHealth solutions in health programs in low-resource settings. The Guide is an interactive, electronic resource that outlines key considerations and resources for planning an mHealth intervention, from concept development and technology design to implementation. Each section provides an overview of key concepts, a summary checklist of considerations, relevant examples, and recommended tools and resources. The guide can be accessed at: www.k4health.org/toolkits/mhealth-planning-guide.

The **mHealth Evidence Database** (www.mhealthevidence.org/) is designed to bring together published reports and articles on mHealth effectiveness, cost-effectiveness, and program efficiency. This collection of resources makes it easier for researchers, program managers, funders, and other key decision-makers to quickly get up to speed on the current state-of-the-art. It includes peer-reviewed and grey literature from high-, middle-, and low-resource settings. Materials are classified using a new mHealth Evidence Taxonomy, developed in coordination with the World Health Organization, and are easily filtered and searched to facilitate the identification of evidence-based, high-impact mHealth practices.

An 8-page brief, **mHealth: Mobile Technology to Strengthen Family Planning Programs**, was recently published as part of USAID's High-Impact Practices in Family Planning series, which identifies mHealth as an emerging practice. This brief highlights evidence in mHealth and family planning programs to date and contains a synthesis of lessons learned for implementation of mHealth programs. The brief can be accessed at: www.fphighimpactpractices.org/resources/mhealth-mobile-technology-strengthen-family-planning-programs

In addition to these 4 tools, another relevant resource is the **mHealth Working Group and website** (www.mHealthWorkingGroup.org). Started in 2009 with 20 members based in Washington, DC, the mHealth Working Group has grown into an international community of over 1,500 members representing more than 450 organizations in 70 countries. The group's mission is to frame mobile technology within a larger global health strategy and to build capacity, encourage collaboration, and share knowledge. Monthly meetings are held in Washington, DC, with invited speakers. Virtual participation in the meetings is also an option. The group's website contains presentations from the monthly meetings along with other resources.

We look forward to seeing more new research related to the roll out and scale up of mobile health applications in *Global Health: Science and Practice*.
